# Cancer Development and Progression in Patients with Heart Failure

**DOI:** 10.1007/s11897-024-00680-y

**Published:** 2024-09-28

**Authors:** Katharina Seuthe, Felix Simon Ruben Picard, Holger Winkels, Roman Pfister

**Affiliations:** 1 Department of Cardiology, Clinic III for Internal Medicine, University of Cologne, Faculty of Medicine and University Hospital Cologne, Kerpener Strasse 62, 50937 Cologne, Germany; 2 Center for Molecular Medicine Cologne (CMMC), University of Cologne, Cologne, Germany

**Keywords:** Heart failure, Cancer, Cardiotoxicity, Cardio-oncology, Reverse cardio-oncology

## Abstract

**Purpose of Review:**

The co-occurrence of heart failure (HF) and cancer represents a complex and multifaceted medical challenge. Patients with prevalent cardiovascular disease (CVD), particularly HF, exhibit an increased risk of cancer development, raising questions about the intricate interplay between these two prevalent conditions. This review aims to explore the evolving landscape of cancer development in patients with HF, shedding light on potential mechanisms, risk factors, and clinical implications.

**Recent Findings:**

Epidemiological data suggests higher cancer incidences and higher cancer mortality in HF patients, which are potentially more common in patients with HF with preserved ejection fraction due to related comorbidities. Moreover, recent preclinical data identified novel pathways and mediators including the protein SerpinA3 as potential drivers of cancer progression in HF patients, suggesting HF as an individual risk factor for cancer development.

**Summary:**

The review emphasizes preliminary evidence supporting cancer development in patients with HF, which offers several important clinical interventions such as cancer screening in HF patients, prevention addressing both HF and cancer, and molecular targets to treat cancer. However, there is need for more detailed understanding of molecular and cellular cross-talk between cancer and HF which can be derived from prospective assessments of cancer-related outcomes in CV trials and preclinical research of molecular mechanisms.

## Introduction

Cancer and heart failure (HF) are the two most significant health burdens globally [1, 2] and their incidences are continuously increasing with a progressively aging population. Historically, much attention was drawn to the risk of HF in patients treated for cancer. While advanced treatment options have reduced cancer mortality, cardiovascular disease (CVD) is the most frequent cause of non-cancer death in cancer survivors, largely due to cardiotoxicity of cancer therapy. The emerging field of Cardio-oncology has traditionally focused on prevention and treatment of cardiotoxicity induced by cancer therapy. However, there is growing evidence supporting a bidirectional relationship between heart failure and cancer. Advanced HF therapies have reduced cardiovascular death leading to an increasing focus of comorbidities for outcome of HF patients, and cancer has been described as the leading cause of non-cardiovascular mortality [1].

Both CVD and cancer share genetic and non-genetic risk factors as well as pathological pathways, including inflammation, oxidative stress, and somatic mutations potentially explaining their coexistence. However, recent studies suggested that CVD and HF in particular causally increase the risk of developing cancer, implementing the term `reverse Cardio-oncology´ [3].

In this review, we focus on the latest epidemiological and clinical findings regarding the incidence of cancer within the HF population. This includes an exploration of common mechanistic pathways and recent preclinical data on HF triggering tumor growth.

## Epidemiological Evidence of Association of Cardiovascular Disease and Cancer

### Cancer in HF Patients

Conflicting clinical data exists on cancer incidence in different HF populations (see Table 1 + 2). Analysis of the Physicians’ Health Study which enrolled 28,341 males showed no association of baseline self-reported HF status with cancer incidence nor cancer related death during a median follow-up of 19.9 years [4]. Moreover, in a Danish national register study of 103,711 individuals aged 30–80 years and diagnosed with HF between 1997 and 2016, the five-year cancer incidence rate remained stable from 1997 to 2016, whereas the overall survival after HF diagnosis significantly increased. Despite higher survival in HF, the risk of cancer did not increase over the years, indirectly suggesting that HF per se might not impact cancer development [5].

**Table 1:** Clinical evidence on heart failure and cancer

Authors (Year)	Study population		Heart failure definition	Follow up time	Number of Patients	Study Design	Findings
Selvaraj et al. (2018)	HF patients from the Physicians’ Health Study	Self-reported Heart failure diagnosis through questionnaire		19.9 years	28,341	Prospective cohort	Found no significant association between HF and the incidence of cancer
Bruhn et al. (2023)	Danish HF patients aged 30–80 years from nationwide registries	First heart failure diagnosis from inpatient or outpatient care from ICD codes		5 years	103,711	Nationwide cohort	Five-year cancer incidence rate remained stable from 1997 to 2016 among HF patients in Denmark
Hasin et al. (2013)	Residents of Olmsted County, Minnesota, with HF diagnosed between 1979 and 2002	HF defined by clinical diagnosis, confirmed by Framingham criteria		7.7 years	961	Retrospective cohort	Demonstrated an increased risk (68%) of cancer in patients with HF
Hasin et al. (2016)	Olmsted County residents post-myocardial infarction with subsequent HF	HF diagnosed post-myocardial infarction, confirmed by clinical records, confirmed by Framingham criteria		4.9 years	1,081	Retrospective cohort	HF post-myocardial infarction has a 71% higher cancer incidence
Banke et al. (2016)	Patients from 26 Danish HF clinics 2002–2009	HF diagnosed by a cardiologist, mainly EF < 45%		4.5 years	9,307	Retrospective cohort	Reported a significantly higher incidence of cancer in CHF patients
Bertero et al. (2022)	HF patients ≥ 50 years from Puglia region in Italy from 2002 to 2018	HF diagnosis in administrative health data		≥ 5 years	104,020	Retrospective community-based cohort from Italy	Showed increased cancer incidence and mortality in patients with pre-existing HF
Roderburg et al. (2021)	HF patients from the Disease Analyser database	HF diagnosis based on ICD codes from the Disease Analyser database		10 years	100,124	Retrospective cohort from German general practices	Found a higher incidence of cancer diagnoses in HF patients
Jaiswal et al. (2023)	Patients with HF from multiple studies included in a meta-analysis	HF diagnosis as per the included studies in the systematic review and meta-analysis			7,329,706 (meta-analysis of 9 studies)	Systematic Review and Meta-analysis	Found a higher risk of incident cancer in HF patients through systematic review and meta-analysis
Sakamoto et al. (2017)	Hospitalised patients with CHF from 2001–2013	HF diagnosed by a cardiologist using the Framingham criteria		4.9 years	5,238	Single-centre retrospective Japanese cohort	Found a four-fold higher incidence of cancer in HF patients
Meijers et al. (2018)	HF patients from the PREVEND cohort	HF diagnosed with NT-proBNP levels		11.5 years	8,592	Community based prospective cohort	Higher baseline levels of NT-proBNP correlated with an increased risk of new-onset cancer
Leedy et al. (2021)	Post-menopausal women 50–79 years in 40 centres, Women’s Health Initiative	HF diagnosis by a trained physician, HFrEF: EF < 50%, HFpEF > 50%		8.4 years	146,817	Prospective multicentre cohort	Found an association between HF and subsequent cancer development, mainly in HFpEF
Malmborg et al. (2018)	Danish residents 30–99 years with myocardial infarction	Myocardial infarction by ICD codes		5–17 years	122,275	Retrospective nationwide Danish cohort	Found increased cancer incidence in patients post-myocardial infarction
Berton et al. (2018)	Patients hospitalized for ACS in four different hospitals	Patients diagnosed for ACS in coronary care unit		17 years	589	Prospective multicentre study	Found a three-fold higher incidence rate for malignant neoplasia after hospitalization for ACS

*HF* Heart failure, *CHF* Chronic heart failure, *ICD* International Statistical Classification of Diseases and Related Health Problems, *HFrEF* Heart failure with reduced ejection fraction, *HFpEF* Heart failure with preserved ejection fraction, *ACS* Acute coronary syndrome

**Table 2:** Cancer entities linked to heart failure

Authors (year)	Clinical Condition	Cancer entities significantly linked to HF	Cancer entities not linked to HF
Hasin et al. (2016)	HF (after myocardial infarction)	respiratory digestive hematologic	
Banke et al. (2016)	CHF	all cancer entities except prostate cancer	prostate cancer
Roderburg et al. (2021)	HF	lip oral cavity pharynx cancers respiratory organ genital organ in females	
Jaiswal et al. (2023)	HF	breast lung haematological colorectal excluded	prostate cancer
Sakamoto et al. (2017)	HF	stomach lung colon breast prostate	
Meijers et al. (2018)	HF with high NT-proBNP levels	female reproductive	
Leedy et al. (2021)	HF with preserved ejection fraction	overall lung colorectal	breast cancer
Berton et al. (2018)	Post-myocardial infarction	overall lung lower urinary tract	
Secretan BL et al. (2016)	Obesity	colon rectum gastric cardia liver gallbladder pancreas kidney oesophageal cancer	
Braithwaite et al. (2012)	Hypertension	breast cancer	
Beak et al. (2016)	Dyslipidemia	prostate breast	
Zhu et al. (2022)	Type 2 Diabetes	colorectal hepatocellular pancreatic	brain buccal cavity oesophageal lung breast urinary bladder laryngeal

*HF* Heart failure, *CHF* Chronic heart failure

However, increasing evidence suggests a contrary perspective. A community based retrospective study of 961 HF patients from Olmsted County showed that HF patients carry a 68% higher risk to develop incident malignancy compared with the normal population during a mean follow-up of 7.7 years, adjusted for body mass index (BMI), smoking and comorbidities [1, 6]. Incident cancer was associated with a 56% excess adjusted mortality risk. In a subsequent prospective study of a different cohort in Olmsted County, 1,081 patients after incident myocardial infarct (MI) were followed over a mean of 4.9 years. Patients who developed HF post MI had a 71% higher incidence of cancer compared with patients without HF after MI. Most common types were respiratory, digestive, and hematologic cancers [7].

In a large Danish cohort of 9,307 outpatients with HF and reduced ejection fraction but no prior cancer diagnosis, the authors found significantly higher incidence rate of various cancer types -excluding prostate cancer- after a 4.5 year follow up when referenced to the general population. Additionally, HF patients with cancer had a higher all-cause mortality rate compared to cancer patients without HF included in the background population [8].

A recent retrospective study involving 104,020 community-based subjects conducted in the Puglia region of Italy revealed a notable increase in both cancer incidence and cancer mortality among patients with HF compared to matched non-HF control subjects, with hazard ratios of 1.76 (95% CI: 1.71–1.81) and 4.11 (95% CI: 3.86–4.38), respectively [9].

Moreover, a retrospective cohort study examined patients with heart failure (n = 100,124) compared to a matched non-heart failure group from 1274 general practices in Germany from 2000–2018 using data from the Disease Analyzer database. Over a follow up of 10 years, 25.7% of HF patients and 16.2% of non-HF patients developed cancer. HF significantly correlated with cancer incidence across various cancer sites, with the highest associations found for lip, oral cavity, and pharynx cancers, respiratory organ cancers, and genital organ cancers in females [10].

These findings were confirmed in a meta-analysis [11] of 9 individual studies comprising 7,329,706 patients. The authors uncovered a significant increase in the incidence of cancer, particularly breast, lung, haematological, and colorectal cancer, in patients with HF compared to those without HF. The large meta-analysis did not show a significant association of prostate cancer incidence with HF.

A Japanese single-centre study compared 5,238 patients with HF who were hospitalized between 2001 and 2013 and followed until April 2015 to a control cohort from a cancer incidence database. The authors reported a four-fold higher incidence of cancer (stomach, lung, colon, breast, and prostate cancer) in the HF cohort in comparison to the control group. In addition, brain natriuretic peptide (BNP) levels correlated positively with cancer incidence, although no correlation was found with left ventricular ejection fraction (LVEF)[12].

In the PREVEND study—a community based prospective, observational cohort with 8,592 participants—Meijers et al. discovered over a median follow-up of 11.5 years that higher baseline levels of N-terminal pro-B-type natriuretic peptide (NT-proBNP), a key marker for HF severity, correlated with an increased risk of new-onset cancer, even after adjusting for age, smoking, and BMI [13]. Notably, higher NT-proBNP levels were significantly linked to the incidence of female reproductive cancers. Moreover, pro-inflammatory cytokines including pro-adrenomedullin, pro-endothelin, and the acute phase C-reactive Protein (CRP) were also associated with an increased risk of cancer. CRP showed the strongest association, indicating that inflammatory processes might mediate the effects of cancer in HF.

Utilizing data from the Women's Health Initiative, Leedy et al. examined cancer incidence in a cohort study of 146,817 postmenopausal women from 1993 to 2015 [14]. This study revealed an association between HF and subsequent cancer development including lung and colorectal cancer, but not breast cancer. In a sub-analysis distinguishing between heart failure with preserved ejection fraction (HFpEF) and heart failure with reduced ejection fraction (HFrEF), HFpEF was significantly linked to an overall higher incidence of cancer, particularly lung and colorectal cancers. In contrast, HFrEF did not show a significant association with overall cancer incidence but was linked to a decreased incidence of obesity-related cancers and an increased incidence of lung cancer. There were no significant differences between HFpEF and HFrEF in overall or site-specific cancer incidence, except for obesity-related and colorectal cancers [14].

Taken together, the majority of studies suggests an association between HF, particularly HFpEF, and diagnosis of certain cancer types, foremost lung and colorectal.

### Cancer in Acute Coronary Syndrome

Coronary artery disease (CAD) is one of the most common causes of HF in industrialized countries, providing a circumscribed pathophysiological pathway also for the association between HF and cancer. The risk of and mortality due to cancer were evaluated in patients who had experienced a MI in a large analysis of four Danish registries (The Danish Civil Registration System registry, the NPR [Danish National Patient Registry], the National Causes of Death Registry, and the Danish National Prescription Registry), which contained information on all Danish residents in 1996 which were followed up until 2012. All age groups of MI patients (n = 122,275) had an increased incidence of cancer (overall, lung and lower urinary tract cancer) and death one year post MI diagnosis [15, 16].

In a long-term prospective study including 589 patients after hospitalization for acute coronary syndrome (ACS), a three-fold higher incidence rate for malignant neoplasia was observed over a 17-year follow-up period in comparison to the general population. Furthermore, patients who developed cancer after ACS were observed to have a poorer prognosis [15, 17].

In summary, although there is conflicting data, most studies suggest a relationship between HF and CVD and incident cancer and worse cancer outcomes. However, it is crucial to acknowledge limitations of this evidence. Most studies included heterogenic cohorts and retrospective analyses, which impair the establishment of causality [18].

Additionally, these studies were often underpowered and not specifically designed to address cancer outcomes in HF patients, i.e. sensitivity and specificity of cancer diagnosis are unclear.

It is important to point out that CVD patients often undergo more intensive medical monitoring compared to the general population. This increased level of surveillance might lead to a higher observed incidence of cancer through detection bias. Frequent diagnostic procedures including lab tests, chest X-rays, CT scans, PET scans, and MRI scans in CVD patients could potentially reveal hidden malignancies in patients [15]. Furthermore, therapies in CVD patients such as anticoagulation may unmask tumor development via tumor associated bleeding complications [19].

Finally, information on HF entities and severity was limited in many studies which might contribute to varying results considering the heterogenous nature of HF and associations with cancer development in subentities. For instance, one study indicated an elevated risk of cancer for HFpEF patients, which potentially might be biased by the extensive extracardiac comorbidities in this population. Consequently, conducting detailed prospective studies is imperative to draw clinically significant conclusions in this domain.

## Co-Occurrence of Cancer and HF

### Shared Risk Factors

**Fig. 1:**
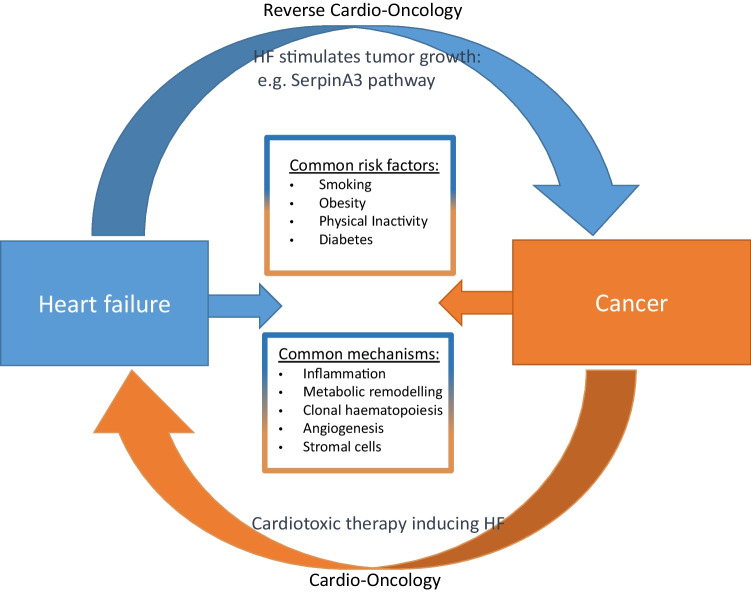
Graphical illustration of the complex bidirectional relationship between heart failure and cancer, *HF* heart failure

The connection between HF and cancer might be also explained through shared risk factors [20], Fig. 1 illustrates the complex relationship between the two conditions. Koelwyn et al. [15] provided a detailed summary on mutual risk factors. The most obvious shared risk factor for CVD and cancer is smoking. Notably, 80–90% of lung cancer related deaths are attributed to smoking. Smoking increases the risk in numerous other cancer subtypes, and approximately 30% of all cancer deaths are smoking-related. Smoking drives cancer development by many mechanisms including direct carcinogenic effects on mutagenesis, epigenetic changes, and inflammation [21]. Concomitantly, smoking is a risk factor for HF via atherosclerosis and CAD [22, 23].

Obese patients, which have a high risk to develop CVD, are also at a high risk to develop cancer. Significant associations between BMI and cancer risk have been reported, especially for colon, rectum, gastric cardia, liver, gallbladder, pancreas, kidney and oesophageal cancer [24]. Obesity is also associated with higher cancer-specific mortality in numerous cancers. Mechanistically, obesity leads to increased levels of various circulating factors such as leptin, glucose, insulin, and insulin-like growth factor 1, thereby contributing to an environment that enhances cell growth and proliferation. These metabolic alterations simultaneously promote chronic inflammation and immune suppression which can contribute to initiation and progression of cancer [25]. This problem appears more prevalent in African Americans compared to other racial groups underlining the complexity and heterogeneity of this relation [26]. Physical inactivity is also linked to an increased risk of cancer and progression. Physical activity benefits cancer prevention and progression through various mechanisms, including immune modulation and metabolic changes [27].

The relationship between hypertension and cancer is less clear, with some studies showing a small increased risk of cancer and mortality (e.g. in breast-cancer) related to high blood pressure [28].

The association of dyslipidemia with cancer is also inconsistent. Increased cholesterol levels are associated with the risk of high-grade cancer entities and cancer recurrence in prostate and breast cancer [29]**.** High cholesterol levels may drive certain cancer types through effects on immune responses and hormone receptors [30].

While extensive research has concentrated on the connection between diabetes and CVD, diabetes is also associated with an elevated risk of cancer. Type 2 diabetes is linked to a 10% increased risk to develop cancer. Notably, higher risks have been observed for specific types, including colorectal cancer, hepatocellular cancer, and pancreatic cancer [31].

The relationship between chronic kidney disease (CKD), a frequent comorbidity of HF, and cancer has gained increasing recognition over the past two decades. This link is bidirectional: certain cancer treatments and nephrectomy in kidney cancer patients can cause nephrotoxicity and CKD, while CKD may lead to cancer through factors like cystic disease, carcinogenic toxins in urine, oxidative stress, and inflammation [32].

Moreover, non-modifiable risk factors common to both cancer and CVD exert influence over the incidence and progression of these conditions. Genetic factors, age, and sex play pivotal roles.

For instance, genetic mutations, particularly in the Wnt/β-catenin pathway and DYRK1B gene, play roles in both CVD and cancer. The Wnt/β-catenin pathway participates in the physical and pathological processes of a variety of organs and diseases [33]. Mutations contribute to CVD development via cardiac hypertrophy, fibrosis and ischemia, while also influencing cancer cell proliferation. DYRK1B mutations are associated with CVD risk factors such as obesity, CAD, hypertension, and diabetes and regulate cellular survival in cancer.

In addition to the above discussed epidemiological associations with clinical risk factors few detailed pathological pathways have been identified over the past years linking cancer and HF and partially explaining their relation [1].

Inflammation plays a significant role in both HF and cancer. Elevated levels of pro-inflammatory cytokines like IL-1β, IL-6, and IL-18 are found in both acute and chronic HF, and these cytokines are also key to malignant transformation and metastasis [34], which often show characteristics of inflamed tissues. IL-1β and IL-6, in particular, are associated with cancer, promoting cancer initiation and progression. In the Canakinumab Anti-Inflammatory Thrombosis Outcome Study (CANTOS), the IL-1β-targeting antibody canakinumab showed positive effects on cardiovascular events and HF hospitalization. Surprisingly, the study hints at a potential decrease in lung cancer incidence and mortality post canakinumab treatment [35].

Lipid mediators like prostanoids, in addition to cytokines and chemokines, play an important role in inflammatory signalling. While their involvement in cancer and CVD is not well understood, elevated prostaglandin E2 levels promote cancer initiation especially in gastrointestinal tumours and suppress immune responses against cancer cells [36]. Moreover, prostaglandin E2 can impact cardiac function by activating gene programs downstream of the prostaglandin E receptor 3 (EP3 receptor) on cardiomyocytes, leading to the secretion of chemokines and induction of chemoattractant signalling [1].

Cancer cells and cardiomyocytes in HF exhibit metabolic reprogramming to adapt and survive under their distinct stress conditions. In both conditions, glucose metabolism plays a crucial role. Cancer cells rely on aerobic glycolysis (Warburg effect) for energy while HF alters the heart's metabolic flexibility, often switching from utilization of fatty acids to glucose [37]. Therapeutic strategies in cancer and HF target these metabolic pathways. Interventions like SGLT2 inhibition in HF and GLUT1 inhibition in cancer showed great clinical promise [38]. The metabolic similarities between cancer and HF offer potential for developing treatments that concomitantly address both conditions.

Moreover, emerging research indicates genetic risk factors as common drivers of both cancer and CVD. Acquired somatic mutations in hematopoietic cells significantly increase the risk of CAD, with the majority of these genetic variants occurring in genes like TET2, DNMT3α, ASXL1, JAK2, and TP53 [1]. These variants provide a competitive growth advantage for mutated hematopoietic stem cells, and subsequently can lead to clonal haematopoiesis and increase the risk of CVD and stroke, possibly through accelerated atherosclerosis as well as HF [39]. Additionally, these genetic variants have been linked to worse outcomes in ischemic heart failure. Understanding how clonal haematopoiesis contributes to atherosclerosis and cardiac dysfunction is essential, and it holds promise for personalized medicine in predicting CV risk and therapeutic responses.

Angiogenesis is also crucial in both HF and cancer. In response to hypoxia, microvascular expansion in the heart is stimulated by the secretion of angiogenic factors like Vascular Endothelial Growth Factor (VEGF) and angiopoietin-1 and -2 [40]. However, this adaptive angiogenic response is suppressed under sustained pressure overload, leading to vascular rarefaction, and contributing to decompensated HF. In the context of cancer, angiogenesis is crucial for tumour growth and dissemination, with new blood vessel formation providing nutrients to cancer cells and facilitating their spread [41]. Drugs inhibiting angiogenesis, such as VEGF inhibitors, have been used in cancer treatment, but their cardiovascular toxicities are potentially severe [42].

In tumours, malignant cells interact with the extracellular matrix (ECM) and stromal cells, promoting growth and invasiveness [43]. Similarly, the heart's ECM and diverse cardiac stromal cells play roles in heart repair and disease [44]. The ECM in both the heart and tumour mediates disease progression and treatment resistance.

Human microvascular pericytes, when transplanted intramyocardially, promote heart recovery post-infarction through paracrine effects. However, blocking pericytes in cancer hasn't shown improvement and could even increase metastasis [41]. Fibroblasts in heart failure and solid tumours undergo similar changes, contributing to tissue remodelling and disease progression. Abnormal fibroblasts share unique molecular signatures, which might be targetable by anti-cancer drugs repurposed for heart failure therapy [45]. This has recently been demonstrated in a preclinical mouse model of heart failure, in which elimination of fibrosis-associated fibroblasts by chimeric antigen receptor T cells (CAR T cells) successfully reduced cardiac fibrosis and improved heart function [46].

Chronic and progressive hyperactivation of the sympathetic nervous system (SNS) and the renin–angiotensin–aldosterone system (RAAS) is a key characteristic of development and progression of HFrEF. Similarly, there is experimental evidence highlighting a potential role of these humoral systems for development of cancer.

The effects of the SNS on cancer are mainly reported via the β-adrenergic receptor (AR) in tumour mouse models [47]. SNS activity may contribute to tumorigenesis via β-AR–dependent activation of stimulatory G protein–protein kinase A and β-arrestin-1 signalling [48], which promotes the accumulation of DNA damage and hampers its repair.

This signalling can contribute to cancer progression by a variety of mechanisms including inflammation, angiogenesis, apoptosis, cell movement, and immune response. In a mouse model of ovarian cancer, Thaker et al. [49] reported that stress-induced catecholamines promoted tumour growth, invasiveness, and vascularization by enhancing the expression of VEGF as the central mediator of neoangiogenesis.

The primary driver of this signalling is norepinephrine within the tumour itself, rather than circulating in the bloodstream [47].

While these effects are well-established in solid tumours, their role in blood cancers is less clear.

Similar experimental evidence exists for the RAAS, which may be particularly relevant via its local organ-specific effects rather than its systemic endocrine effects [50].

In vitro and in vivo studies have shown that the type 1 angiotensin receptor (AT1R) promotes the growth, vascularization, and invasiveness of various cancers. Furthermore, in human ovarian carcinoma samples, AT1R levels were positively correlated with VEGF expression and tumour vessel density [51]. Gene silencing or pharmacological inhibition of AT1R in animal models has been found to reduce tumour vascularization and macrophage infiltration. Additionally, genetic variations in RAAS components, such as the high-activity or genotype of the angiotensin-converting enzyme polymorphism, may increase cancer risk [52]. Aldosterone has been involved in promoting cancer metastasis, while its blockade or reduced synthesis prevented the dissemination of tumours in animal models.

Taken together, although these data provide a plausible pathophysiological link between heart failure and cancer development, it is unclear so far how relevant these mechanisms are in the clinical context.

## Effects of HF Pharmacotherapies on Cancer

### Beta-blockers (BB)

Due to the above-mentioned link between SNS and cancer, beta-blockers have been explored for their potential to decrease cancer incidence and improve cancer outcomes.

Beta-blockers have been extensively studied for their potential anti-cancer effects, particularly in preclinical work. Most studies have focused on the non-selective beta-blocker propranolol, which has shown significant anti-cancer effects in vivo, including the inhibition of tumour growth, reduction of metastasis, modulation of the tumour microenvironment, and suppression of angiogenesis e.g. in melanoma models [53]. Further studies suggest that the effects of propranolol are amplified when combined with other therapies [54].

Other beta-blockers, such as carvedilol, have also demonstrated significant anti-cancer effects in various cancer types when used alone. Studies on beta1-selective blockers like metoprolol and nebivolol, as well as the non-selective labetalol, have shown either direct anti-cancer effects or an ability to enhance the efficacy of other cancer treatments.

Propranolol and beta2-adrenoceptor blockers generally showed stronger anti-cancer activity compared to beta1-selective blockers [55].

Despite promising preclinical results, clinical studies have yielded inconsistent findings. Some meta-analyses show no significant effect of beta-blockers on cancer risk [56] while others report mixed results depending on the cancer type. For instance, beta-blockers use has been linked to an increased risk of melanoma and kidney or bladder cancer in some studies, while it appears to reduce the risk of hepatocellular carcinoma in patients with liver cirrhosis. Regarding cancer outcomes, the impact of beta-blockers is similarly inconsistent, with some studies showing improved outcomes in certain cancers, like breast cancer, and others showing no benefit or even worse outcomes, as seen in some lung cancer studies. The complexity of beta-adrenoceptor selectivity and its influence on cancer outcomes further complicates the interpretation of existing study results, highlighting the need for more research to clarify these relationships.

### RAAS- Inhibition

As stated above, research suggests that dysregulation of RAAS may promote cancer, primarily through the AT1R signalling [50]. This led to investigations into the potential anti-cancer effects of RAAS inhibitors, such as angiotensin-converting enzyme inhibitors (ACEIs) and angiotensin receptor blockers (ARBs).

Pre-clinical in vivo studies mostly show beneficial outcomes, for RAAS inhibitors either on their own or in combination with other anti-cancer therapies*.* However, contradictory results were reported in an early study by Wysocki et al. [57] where captopril did not show significant anti-cancer effects and was associated with increased mortality in immunocompetent mouse models of renal cancer. In contrast, a more recent study using a similar cancer model and the same cell line found that captopril significantly reduced primary tumour weight and lung metastases, with treatment starting two days before tumour inoculation at a lower dose [58]. It remains unclear whether these findings represent a class effect, as most studies have focused almost exclusively on captopril. A comprehensive, systematic research strategy to assess the effects of different ACEIs is still needed.

ARBs inhibit the AT1R, the primary target of angiotensin II, the main effector of the RAAS. In tumour-bearing mice, ARBs have shown significant anti-cancer effects by reducing tumour growth, fibrosis, metastases, neo-angiogenesis, and by modulating the tumour immune microenvironment. A key study by Rhodes et al. [59] found that AT1R is overexpressed in 10%–20% of breast cancer cases, particularly in a subset of oestrogen receptor-positive, ERBB2-negative breast cancers, suggesting potential benefits from ARB therapy, especially losartan. However, other studies have reported contradictory findings, with some in vitro and in vivo studies showing little or no anti-cancer effects from ARBs, particularly losartan or irbesartan.

The potential impact of RAAS blockade on cancer in the clinical setting was explored in a meta-analysis [60] focusing solely on randomized controlled trials (RCTs) of ARBs. This analysis found a significant association between ARB use and overall cancer risk, particularly linked to new-onset lung cancer. However, concerns were raised about the reliability of these findings due to inconsistent cancer diagnosis adjudication across the included studies.

Other meta-analyses of RCTs reported no significant link between ARB or ACEI use and cancer risk compared to placebo. Similarly, meta-analyses of cohort studies suggested no association between RAAS inhibitor use and cancer incidence across various cancer types. However, some non-randomized studies indicated a reduced incidence of oesophagus, colorectal, prostate, and lung cancers, but an increased risk of renal cancer and melanoma among ACEI/ARB users compared to non-users [55].

Mineralocorticoid receptor antagonists (MRAs) have shown some promise in reducing tumour volume and inhibiting metastasis in preclinical in vivo studies [61]. Spironolactone [62] had superior anti-cancer efficacy compared to eplerenone, though the underlying mechanisms require further exploration. Yet, spironolactone showed no anti-cancer effects in liver and pituitary cancer cell lines. Additionally, research by Aldaz et al. [63]and colleagues demonstrated that spironolactone, whether used alone or with dexamethasone, protected glioblastoma cells from radiation-induced damage.

### ARNI

There is a lack of substantial preclinical and clinical evidence on the effects of angiotensin receptor-neprilysin inhibitors (ARNIs), specifically sacubitril/valsartan, on cancer. While ARNIs are beneficial in treating HF by enhancing the effects of endogenous natriuretic peptides (NPs), their impact on cancer remains unclear. In a key RCT, cancer-related deaths were similar between patients using ARNIs and those using ACEI [64].

NPs have demonstrated tumour-inhibitory effects in several in vitro and in vivo studies [55]. However, these findings should be approached with caution, as some cancer cells can also produce NPs, which raises concerns about the generalizability of their anti-tumour effects. Additionally, neprilysin inhibition, a mechanism of ARNIs, increases the availability of various peptides that may affect cancer cell biology, necessitating further research to fully understand ARNIs' role in cancer progression.

### SGLT-2 Inhibitors

Research into sodium-glucose cotransporter 2 inhibitors (SGLT2Is) suggests they might have potential anti-cancer effects due to their ability to block glucose uptake, which is crucial for cancer cell survival [38]. Initial studies demonstrated SGLT2 expression in human pancreatic and prostate cancer cells and showed that SGLT2Is could reduce tumour growth in a pancreatic cancer model. Preclinical studies further supported these findings, showing that SGLT2Is inhibit cancer progression by mechanisms like activating adenosine monophosphate-activated protein kinase (AMPK), inhibiting mTOR, reducing inflammation, and affecting other pathways like the Hippo signalling pathway [65].

However, experimental evidence does not translate into clinical observations. In safety trials of SGLT2Is for diabetic patients, no significant increase in overall cancer events was found, though a slight rise in bladder cancer in men and breast cancer in women was noted among those treated with SGLT2Is [66].

A recent meta-analysis suggested a reduced overall cancer risk with SGLT2I use, particularly with dapagliflozin and ertugliflozin, though this finding may have been skewed by a few large trials [67]. Earlier meta-analyses showed mixed results, with some indicating increased cancer risks, especially for bladder cancer with empagliflozin [68] and in obese patients with longer follow-up periods. However, other studies reported no significant changes in risks for cancers like breast, lung, prostate, and melanoma [69].

The use of SGLT2Is in HF patients is relatively new, and most current data are derived from diabetic populations. The effects of SGLT2Is on cancer outcomes in HF patients require further investigation.

### Diuretics

Interestingly, the Na–K-2Cl transporter, the target of loop diuretics, is also expressed on cancer cells and plays a crucial role in their growth. Preclinical studies have shown that furosemide may have anti-cancer effects by inhibiting this transporter. However, these effects have not been observed in clinical studies[70]. Similarly, while thiazide diuretics like hydrochlorothiazide are not primarily used for decongestion in HF, some studies have suggested a potential link between their use and an increased risk of skin cancer, though a recent meta-analysis found no such association[71]. The relationship between diuretics and cancer, particularly in HF patients, remains unclear.

## Preclinical Data on HF and CVD and Tumor Growth

While shared risk factors contribute to the association of HF and cancer, recent preclinical studies suggest that HF and CVD can directly stimulate tumour growth and therefore might be identified as an individual risk factor.

In 2018, Meijers et al. were the first to establish a crosstalk between heart failure and cancer growth in a preclinical model [13]. Particularly, MI induced HF enhanced polyp formation in the APC^min^ colon cancer mouse model. Permanent ligation of the left anterior descendent coronary artery for six-weeks induced increased polyp volume and mass, which correlated with the degree of MI-induced left ventricular fibrosis and reduction in left ventricular ejection fraction (LVEF). Transplantation of infarcted hearts one-week post-surgery into the cervical region of healthy recipient APC^min^ mice led to increased polyp numbers and mass in the APC^min^ recipient, which was not observable in control mice or APC^min^ mice transplanted with sham surgery hearts. These findings suggest that accelerated cancer progression was not due to hemodynamic changes in the failing heart, but the secretome of the failing heart itself [13]. Notably, LV fibrosis and LVEF correlated also with increased cancer cell proliferation as well as tumour volumes in the transplantation model, highlighting that the absolute volume of infarcted tissue and therefore potential source of pro-oncogenic factors released into the circulation are the main factors behind this cardio-oncogenic crosstalk. Cross-referencing the animal model and plasma protein levels of 101 patients and 180 sex and age matched controls showed upregulation of Ceruloplasmin, Fibronectin, Paraoxonase1, SerpinA1, and SerpinA3 as potential factors behind the observed tumorigenic effect. In vitro stimulation of the human colon cancer cell line HT29 identified SerpinA3 as a driver of cancer cell proliferation via AKT pathway activation, highlighting the protein SerpinA3, which is released from failing hearts, as a potential driver of colon cancer tumorigenesis. Notably, SerpinA3 has been implicated as an acute phase protein during immune reactions in CVD, neurological diseases [72] and as mediator of both cancer progression and immune suppression in the tumor microenvironment [73].

Yuan et al. [74] reported that increased glioma expression levels of SerpinA3 correlated with low immune cell infiltration into the tumour tissue and a poor survival prognosis. Furthermore, SerpinA3 has been implicated in tumour invasiveness and increased epithelial to mesenchymal transition (EMT) via regulation of EMT markers E-cadherin, N-cadherin vimentin and EZH2 in an in-vitro model of triple-negative breast cancers [75]. Recently, Caller et.al. identified that extracellular vesicles released from cardiac mesenchyma stromal cells post MI were able to accelerate tumour growth in heterotopic and orthotopic lung tumour models [76].

Cancer and HF share further mechanisms including immune responses. Recently, the importance of innate immune responses as drivers of tumour growth post MI was highlighted by Koelwyn et al. [77]. The authors observed accelerated tumour growth and cell proliferation at the tumour border in tumours derived from syngenically implanted E0771 cells in mammary fat pads of C57BL6J mice post MI surgery in comparison to sham controls. These findings were corroborated in the spontaneous breast cancer mouse model MMTV-PyMT, which showed accelerated tumour growth and increased lung metastasis formation post MI [77]. Immune cell analysis revealed increased numbers of CD11b^+^ Ly6C^high^ monocytes in tumours post MI in comparison to sham operated animals, a cell population that has been well described to systemically increase in numbers post MI and extravasate into the infarcted heart [78]. CD11b^+^ Ly6C^high^ monocytes are highly plastic and shaped by their respective environment. They can be either pro-inflammatory in the context of an acute immune reaction, but immunosuppressive in a predominantly immunosuppressive environment, like the TME [79].

Koelwyn et al. observed increased levels of anti-inflammatory and immune-suppressive regulatory T cells (Tregs) and CD11b^low^MHCII^high^ tumour-associated macrophages, which largely depend on CD11b^+^ Ly6C^high^ monocytes, in the TME, of MMTV-PyMT mice post MI surgery [77]. Depletion of CD11b^+^ Ly6C^high^ monocytes post diphteria toxin injection in a CCR2-dependent diphteria toxin receptor (CCR2-dtx) transgenic mouse model rescued the effects of MI onto tumour growth, metastasis formation and immunosuppression.

In extension of HF by surgically induced myocardial infarction, Avraham et al. [80] demonstrated that early-stage cardiac remodelling caused by overload-induced cardiac hypertrophy in a transverse aortic constriction (TAC) model drives tumorigenesis in a similar manner. TAC surgery induced pro-tumorigenic environment in female and male wildtype C57BL6/J mice transfected with PyMT cancer cells into the mammary fatpads (female) or LLC lung cancer cells subcutaneously (male). Both models showed an increase in tumour growth, assessed by KI67 staining and absolute tumour mass by TAC in comparison to sham controls. Furthermore, periostin levels increased in TAC-operated mouse plasma. Notably, periostin induced PyMT cell proliferation in vitro. Awwad et al. [81] corroborated the finding in a non-surgical mouse models of heart failure utilizing a cardiomyocyte-specific activating transcription factor 3 (ATF3) overexpression mouse model. ATF3 is a transcription repressor that is increased by multiple stressors and growth stimuli and has been elevated in HF patients and in mice post TAC-surgery mediated HF [82]. Transgenic ATF3 overexpression increased plasma ceruloplasmin, CTGF and fibronectin levels, which exert known pro-tumourogenic functions. ATF3-transgenic mice had decreased cardiac function and enhanced tumour growth and lung metastasis formation post PyMT cancer cell transfer. These studies underline a direct crosstalk between the failing heart and the growing tumour, which involves soluble and cellular mediators. However, the intricate cross-talk is not well-understood and presents an exciting new research field. A better understanding of the cross-talk between the heart and cancer is urgently needed to design better and more specific anti-cancer drugs, which will preserve heart function. An overview of the current preclinical literature is given in Table 3.

**Table 3:** Preclinical evidence of heart failure and cancer

Authors (Year)	Title	Study Design	Experimental model	Findings
Meijers et al. (2018)	Heart failure stimulates tumour growth by circulating factors	Experimental study (animal model) + Observational study	LAD myocardial infarction model, APC^min^ tumor model, inVitro HT-29 culture, Plasma analysis of human HF patients and healthy controls	Severity of LV dysfunction and fibrotic scar correlate with tumour growth and elevated protein levels of serpinA3, A1, fibronectin and ceruloplasmin. SerpinA3 is shown to drive proliferation in HT-29 cancer cells
Fissolo et al. (2021)	CSF SERPINA3 Levels Are Elevated in Patients with Progressive MS	Experimental study (animal model) + Observational study	Transcriptional + proteome analysis of EAE, Candidate biomarker validation by ELISA from MS patients (n = 65) and healthy individuals (n = 30)	Elevated SERPINA3 and S100A4 levels in murine EA and the cerebrospinal fluid of progressive multiple sclerosis patients
Yuan et al. (2021)	Highly expressed of SERPINA3 indicated poor prognosis and involved in immune suppression in glioma	Experimental study	Meta analysis of Chinese Glioma Genoma Atlas databases, validation of predicitions by TMA and RNAscope from human gliomas (n = 321)	High levels of SERPINA3 expression are linked to poor prognosis and immune suppression in glioma patients, driving both tumor cell proliferation and reduction of immune cell infiltration
Zhang et al. (2021)	Overexpression of SERPINA3 promotes tumor invasion and migration, epithelial-mesenchymal-transition in triple-negative breast cancer cells	Experimental study	Meta analysis of TCGA and GEO databases, InVitro analysis of TNBC cells lines MDA-MB-321, BT549 & MDA-MB-436, wound healing & transwell assays	SERPINA3 overexpression enhances tumor invasion, migration, and epithelial-mesenchymal transition in triple-negative breast cancer cells
Koelwyn et al. (2020)	Myocardial infarction accelerates breast cancer via innate immune reprogramming	Experimental study (animal model)	LAD myocardial infarction model, Murine mammary cancer cells line E0771, Transgenic mouse model MMTV-PyMT, Bone marrow transfer & monocyte depletion	MI accelerates breast cancer progression through epigenetic reprogramming of Ly6Chigh monocyte reservoirs in the bone marrow to immunosuppressive and increased Ly6Chigh monocyte recruitment into circulation
Källberg et al. (2012)	CD11b + Ly6C + + Ly6G- cells show distinct function in mice with chronic inflammation or tumour burden	Experimental study (animal model)	In vitro coculture and t-cell suppression models utilizing CD11b^+^Ly6C^++^Ly6G^+^ cells sorted from spleen, tumor tissue or inflammatory granulomas	CD11b + Ly6C + + Ly6G- cells display distinct functions (MDSC, pro-Inflammatory) depending on chronic inflammation or tumour burden, influencing disease progression
Avraham et al. (2020)	Early Cardiac Remodeling Promotes Tumor Growth and Metastasis	Experimental study (animal model)	Transverse aortic constriction mouse model	Early cardiac remodelling caused by TAC contributes to tumour growth and metastasis through elevated periostin levels, causing alterations in the tumour microenvironment
Awwad & Aronheim (2022)	Cardiac Dysfunction Promotes Cancer Progression via Multiple Secreted Factors	Experimental study (animal model)	Heart hypertrophy induced by ATF3 overexpression murine model, In vitro tumor cell culture supplemented by ATF3 mouse serum, cytokine array	Cardiac dysfunction accelerates cancer progression by releasing multiple factors that influence tumor growth and immune responses
Zhou et al. (2011)	Activating transcription factor 3 deficiency promotes cardiac hypertrophy, dysfunction, and fibrosis induced by pressure overload	Experimental study (animal model)	Heart hypertrophy induced by ATF3 overexpression murine model, ATF banding, neonatal mouse cardiomyocyte culture, histology of human HF patient hearts	Deficiency in activating transcription factor 3 leads to exacerbated cardiac hypertrophy, dysfunction, and fibrosis under pressure overload conditions
Caller et al. (2024)	Small Extracellular Vesicles From Infarcted and Failing Heart Accelerate Tumor Growth	Experimental study (animal model)	LAD myocardial infarction model, cMSC culture, sEV analysis, hetero- and orthotopic murine lung and breast cancer model, intervention model: sEV depletion and spironolactone treatment	Failing hearts (MI) release higher amounts of sEV bearing proneoplastic factors, enhancing tumour growth and neoplasia

*MI* Myocardial infarction

## Future Clinical Perspectives

Following recent first guidelines on cardio-oncology the awareness of healthcare providers on the important role of detecting and monitoring cardiac disease during or after cancer treatment slowly increases. The use of artificial intelligence (AI) is being explored to improve the precision and accuracy of cardiac assessments, such as LVEF and global longitudinal strain (GLS), in the context of cardio-oncology. AI has the potential to enhance the prediction and early detection of cardiovascular adverse events in patients undergoing cancer therapy [83].

However, the opposite direction-detection of cancer in patients with HF – is largely under recognized in the clinical community. A first approach is specialized clinics like CHIP (Clonal Haematopoiesis of Indeterminate Potential). These clinics are dedicated to the evaluation and management of patients with clonal haematopoiesis, a condition that increases the risk of developing both hematologic cancers and CVDs. CHIP clinics employ a multidisciplinary approach, bringing together haematologists, cardiologists, genetic counsellors, and other specialists to closely monitor patients, provide lifestyle guidance, and intervene early if signs of disease progression are detected. However, the clinical evidence supporting the pathophysiological link between HF and HF therapy and development and progression of cancer currently seems too weak to deduce direct clinical consequences. Future research is needed with prospectively monitoring patients with cardiovascular disease and heart failure with respect to occurrence of cancer in large populations as a basis for an epidemiological characterization of both diseases.

## Conclusion

While the causal effect of cancer and cancer therapies on development of HF is well established, the role of HF as an independent risk factor for cancer is less clear. Several studies indicate a higher incidence of cancer and worse cancer prognosis in HF and post-myocardial infarction patients. This correlation is partially attributed to shared general risk factors and highlights the large potential of preventive interventions for both diseases in the context of public health efforts. Emerging preclinical data suggest pathophysiological pathways linking tumor growth with HF, implying HF as an independent risk factor, and providing future targets for therapeutic interventions in order to improve prognosis of HF patients.

## Data Availability

No datasets were generated or analysed during the current study.
